# Supplementary material for: Neuronal detection triggers systemic digestive shutdown in response to adverse food sources in Caenorhabditis elegans
Source: eLife. 2025 Oct 3;14:RP104028. doi: 10.7554/eLife.104028 (PMC12494379; doi:10.7554/eLife.104028)
Supplement: Figure 3—figure supplement 1—source data 1. [file elife-104028-fig3-figsupp1-data1.zip › Figure 3—Figure Supplement 1-Source Data1/Figure 3—Figure Supplement1.pdf]

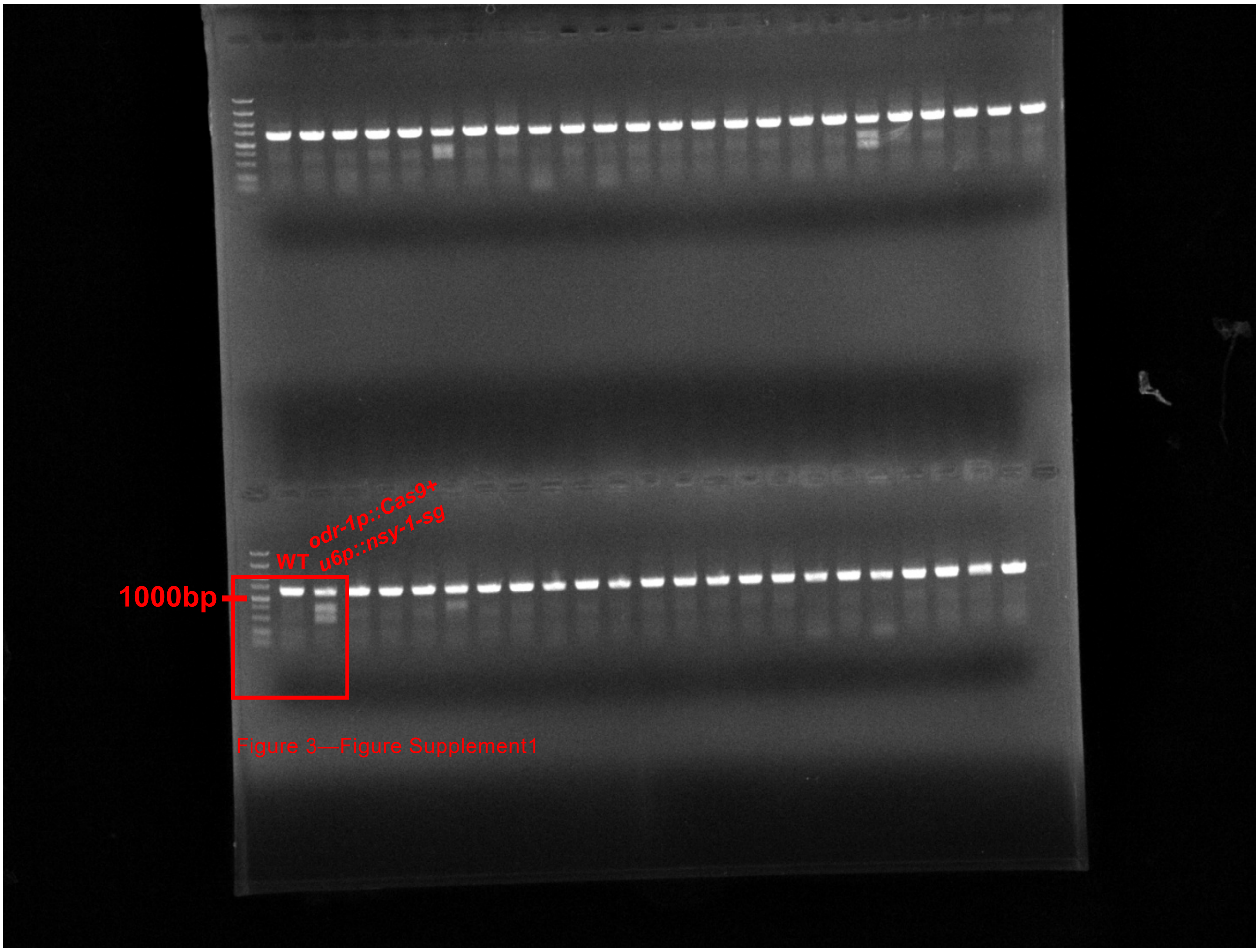

Figure 3—Figure Supplement1
