# Supplementary figures and images for: Neuronal detection triggers systemic digestive shutdown in response to adverse food sources in Caenorhabditis elegans
Source: eLife. 2025 Oct 3;14:RP104028. doi: 10.7554/eLife.104028 (PMC12494379; doi:10.7554/eLife.104028)

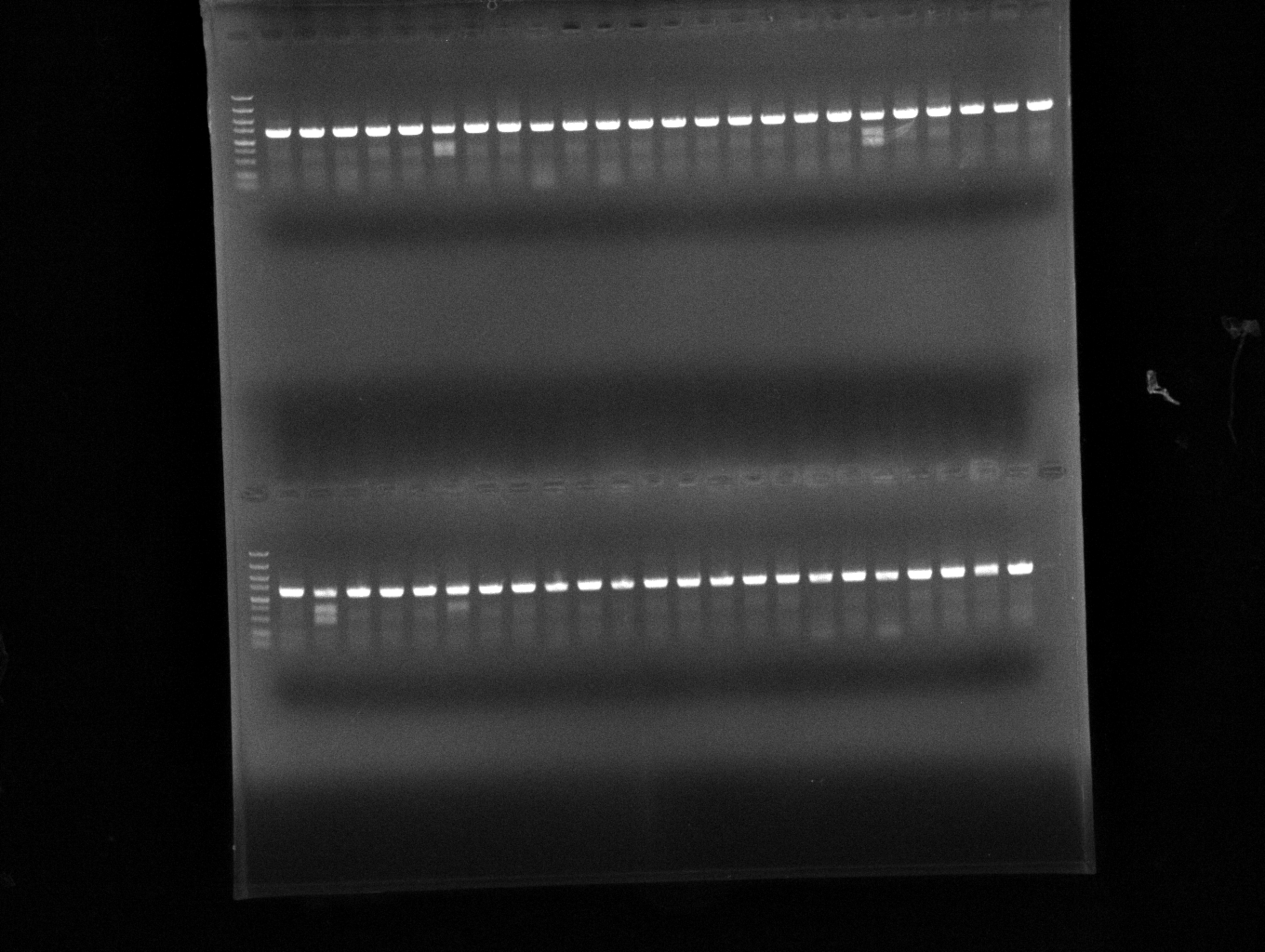

Supplement: Figure 3—figure supplement 1—source data 2. [file elife-104028-fig3-figsupp1-data2.zip › Figure 3—Figure Supplement 1-Source Data2/Original gels.tif]
